# The Frequency and Risk Factors Identified for HBsAg and Anti-HCV in a Preoperative Screening of Patients Admitted in the Surgical Department at Al-Tibri Medical College Hospital

**DOI:** 10.7759/cureus.8938

**Published:** 2020-07-01

**Authors:** Sadaf Jabeen Malik, Rekha Melwani, Farhat Bano, Bilal Suria, Ihsanullah Sial, Bushra Tasneem, Madiha Ariff

**Affiliations:** 1 Surgery, Al-Tibri Medical College, Isra University, Karachi, PAK; 2 Surgery, Shaheed Muhtarama Benazir Bhutto Medical College, Karachi, PAK; 3 Urology, Al-Tibri Medical College and Hospital, Isra University, Karachi, PAK; 4 Surgery, Al-Tibri Medical College and Hospital, Isra University, Karachi, PAK; 5 Surgery, Abbasi Shaheed Hospital, Karachi, PAK; 6 Internal Medicine, Dow University of Health Sciences, Karachi, PAK

**Keywords:** preoperative screening, risk factors, hepatitis

## Abstract

Objective

The aim of this study is to determine the frequency and risk factors for hepatitis B surface antigen (HBsAg) and anti-hepatitis C virus (Anti-HCV) in a preoperative screening of patients admitted at the surgical unit of Al-Tibri Medical College and Hospital and Lyari General Hospital, Karachi.

Materials and methods

This study was conducted at the surgical units of Al-Tibri Medical College Hospital and Lyari General Hospital, Karachi. This study was conducted for six months from 30th June 2017 to 31st December 2017. This observational study was performed using a non-probability convenient sampling technique. All patients who were undergoing elective and emergency surgical procedure were selected for the study and screened for HBsAg and anti-HCV by immunochromatographic test (ICT) method at the hospital laboratory. The risk factors identified as parenteral injections, past surgical procedure, blood transfusion, etc. along with demographic data as age, gender, and district residential status were recorded on a specially designed proforma. Data were analyzed using Statistical Package for the Social Sciences (SPSS) version 23. Descriptive statistics were applied, and the qualitative data were expressed as the mean and standard deviation.

Results

Among a total of 360 patients included in the study, 63 (21.38%) were found with anti-HCV positive, and 14 (3.88%) were HBsAg positive with none of the patients found to have co-infection. The male-to-female ratio was 2.3:1. The mean age of patients was 32.34±4.3 years. The age range affected commonly in the study population was 21-30 years, 24(31%). The commonest risk factor for transmission of viral infection was parenteral injection abuser 31(40.2%), followed by surgical procedure 13(18.1%), blood transfusion 10(12.9%), and barber shave 7(9.1%).

Conclusion

It was predicted in our study that hepatitis C was more common in patients screened preoperatively for surgery. However, the most frequent risk factor in these patients was parenteral abuse history. The preoperative screening is an important investigative tool for the identification of silent cases in the community for preventive measures adoption and treatment for silent carriers.

## Introduction

Hepatitis, by definition, is inflammation of the liver, which if untreated or undiagnosed causes shrinkage of the liver, leading to functional devastating consequences on the human body. Hepatitis B and C are the leading causes of liver diseases, and the World Health Organization (WHO) estimates a 3% prevalence of hepatitis C virus (HCV) worldwide [1]. The annual mortality owing to hepatitis B virus (HBV)-related acute and chronic liver disease is estimated to be 5.2 million. In a developing country like Pakistan, poverty and lack of resources and social awareness with dangerous, unhealthy practices account for the enormous burden of chronic Hepatitis B and C-related liver diseases. The prevalence of Hepatitis B is 10% and is 7% for Hepatitis C [2].

The major causative factors of chronic liver disease are hepatitis B and C, with both being the most common public health issues in society [3]. Recent records reveal that 175 million people have hepatitis C virus. Globally, around 500,000 to 1000,000 HBV-induced mortalities have been noticed yearly owing to hepatocellular carcinoma and cirrhosis [4]. The Centre for Disease Control and Prevention of the United States of America concludes that 2.7 million population is suffering from hepatitis C-induced comorbidities, affecting 40% of patients with chronic liver diseases; these alarming statistics focus on the increased need for liver transplantation [5-6].

Hepatitis B virus (HBV) is frequently found among children and young Asian population [7]. In Pakistan, HBV is found in the middle zone with an incidence rate of 3 to 4%. However, HCV and HBV have become a prominent health issue [8]. In general, all the age group populations are affected, as the potential risk factors are widespread with ignorance and poverty, especially in the rural areas in our country, as our study population primarily includes rural dwellers of poor socioeconomic class. This study is an addition to the local research studies in Pakistan conducted to identify silent seropositive patients on preoperative screen; over the years, up to 25% increase has been observed in the semi-urban population [9]. The most significant risk factors are injurious use of unsterilized syringes, history of blood transfusion, and surgical procedures [10]. The prevalence of Hepatitis C is as high as 20.8% in a healthy and high-risk population, sharing syringes as evident in a study conducted at Quetta in apparently healthy blood donors [11]. 

The emergence of seropositive patients is a serious community health issue, and identification is important in preoperative time to follow strict safety precautions in theatre for prevention of spread. The objective to carry out the study at teaching hospitals was to emphasize preoperative screening in minor and major cases with strict theatre precautions and directed for further treatment, as silent cases as the first study of such type at both teaching institutions. This study aimed to screen in pre-operative time patients for hepatitis B surface antigen (HBsAg) and anti-HCV positive by immunochromatographic test (ICT) method along with risk factors for the disease.

## Materials and methods

This cross-sectional descriptive study using a non-probability purposive sampling technique was conducted in the surgical department of Al-Tibri Medical College and Hospital and Lyari General Hospital, Karachi. This study was conducted for six months, from 30th June 2017 to 31st December 2017. The ethical approval for the study was taken from the Institutional Research and Ethical committee. Verbal informed consent was taken from the patients or their guardians.

A total of 360 patients were selected during the study period, and they were admitted for elective and emergency surgical procedures. All patients aged >5 years, who are not known to be anti-HCV and HBsAg seropositive previously, were included in the study. Chronic carriers or patients on antiviral treatment were excluded from the study. All the cases were preoperatively screened for HBsAg and anti-HCV. Hepatitis B virus and HCV screening are very common and mandatory in tertiary care hospitals as a pre-operative investigative tool for the assessment of their incidence as well as to adapt well preventive measures before surgery. Serology was performed and evaluated by following standard operating procedures. Rapid ICT for qualitative assessment of hepatitis B surface antigen and detection of antibodies for hepatitis C was performed as a screening technique in the study. The cases that were reactive to the rapid method kit were further reassessed using enzyme-linked immunosorbent (ELISA). The cases which were found positive on ELISA were included in the study. The risk factors identified on specially designed proforma were parenteral injections, past blood transfusion, barber shave, and surgical procedures. Proforma for data collection was designed and kept in the ward, and doctors were assigned as part of the research, who collected data for risk factors and documented. The data collected were analyzed using the Statistical Package for Social Sciences (SPSS) Version 23. The quantitative data were reported as the frequency in percentages, and results were generated.

## Results

A total of 360 patients were selected for the study by strictly following the inclusion criteria. All of these subjects were screened for anti-HCV and HBsAg by ICT. Out of these, 63 patients were anti-HCV positive and 14 were HBsAg positive (Table 1), while none of the patients had co-infections. The mean age of patients was 42.42±7.58 years with a male-to-female ratio was 2.3:1.

**Table 1:** Status of anti-HCV and HBsAg seropositive cases (n = 360) *ICT= Immunochromatographic testing +Hepatitis B surface antigen ++Anti-Hepatitis C virus ICT, immunochromatographic test

ICT Testing*	n (%)
HBsAg^+^	14 (3.88%)
Anti-HCV ^++^	63 (21.38%)
Negative	283 (74.74%)
Total	360 (100%)

The patients were categorized based on five age groups, and their frequency and percentage were reported. The groups included age groups between 21-30, 31-40 years, 41-50 years, >50 years, and <20 years. The mean age of patients was 32.34±4.3 years. The commonest frequency observed was in the age group of 21-30 years for both viruses in 24 (31.1%) cases. The minimum frequency was found in the last age group of <20 years with 9 (11.6%) cases having only HCV (Table 2). 

**Table 2:** Age distribution for seropositive patients (n = 77) +Hepatitis B surface antigen ++Anti-Hepatitis C virus

Variable		HBsAg^+^ n (%)	Anti HCV^++^ n (%)
Age groups	<20 Years	0 (0.0%)	9 (14.28%)
	21-30 Years	7 (50.0%)	17 (26.98%)
	31-40 Years	5 (35.7%)	14 (22.22%)
	41-50 Years	2 (14.29%)	13 (20.63%)
	>50 Years	0 (0.0%)	11 (17.46%)
	Total	14 (18.8%)	63 (81.8%)

**Figure 1:**
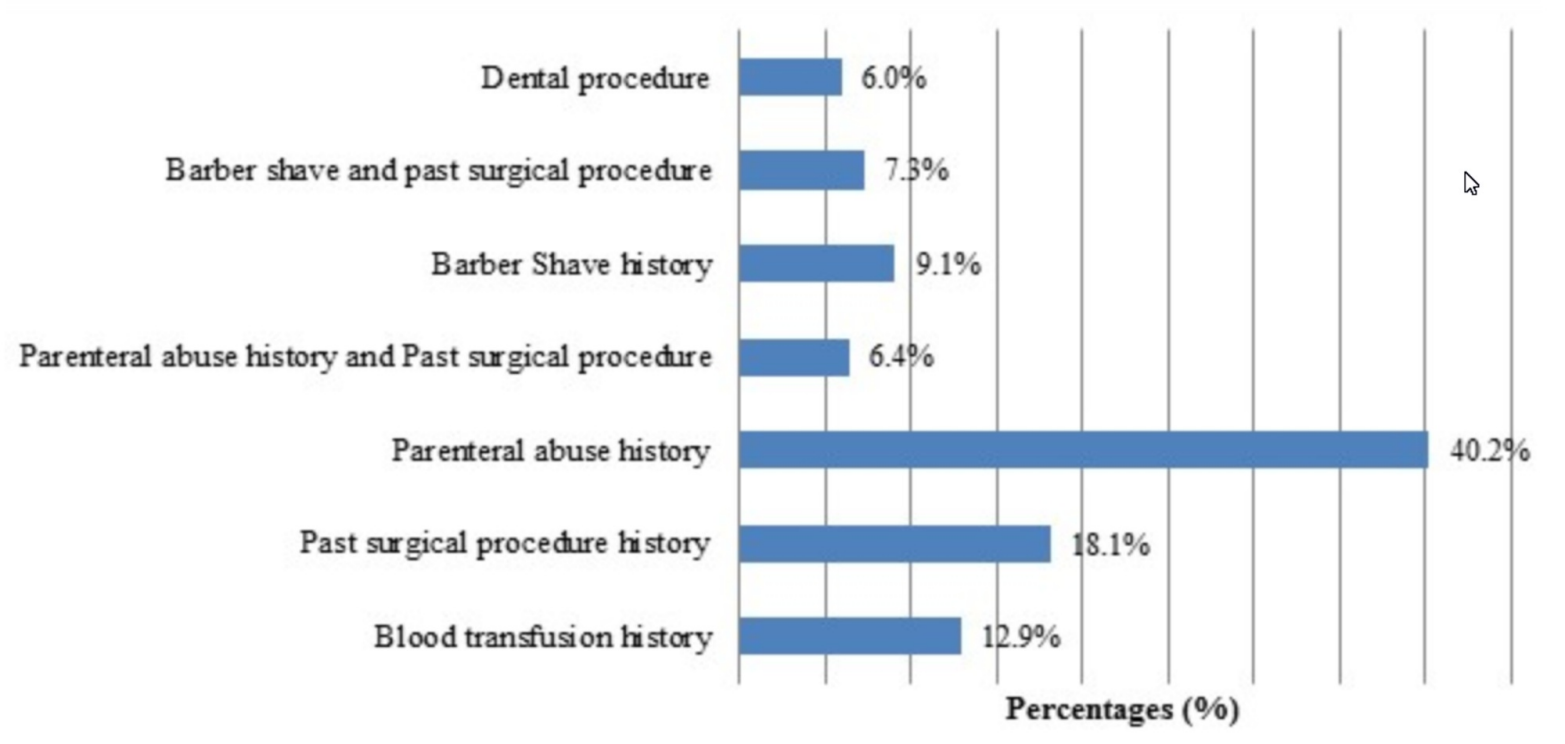
Frequency distribution for risk factors (n = 77)

Data regarding risk factors were recorded as follows: 10 (12.9%) cases had a history of blood transfusion, 13 (18.1%) patients had past surgical procedures, 31 (40.2%) subjects had an addiction of parenteral abuse, 7 (9.1%) cases had a history of barber shave, 5 (6.4%) cases had parenteral abuse as well as past surgical procedure history, 6 (7.3%) cases had a history of barber shave and past surgical events, and 5 (6%) subjects had a history of past dental surgery (Figure 1).

## Discussion

Viral hepatitis cases, in both acute and chronic carrier forms, are increasing in number, worldwide [12]. The prevalence of Hepatitis B and C-associated liver diseases is higher in the underdeveloped world due to the lack of awareness and immature health practices, contrary to the industrialized western world; the low burden of disease is mainly due to immigrants and chronic carriers [13]. Hepatitis B carrier rate in Pakistan is 3-4%, and 10 million Pakistani populations are Hepatitis C carriers. In this study, the frequency and male-to-female ratio are comparable to a local study done by Rafique *et al*. [2].

Our study found more male patients (53.2%), compared to females (46.8%), being infected with the virus, as validated by the Eastern literature, which shows that males from the rural and lower socioeconomic classes were seropositive for Hepatitis C [14]. Our study population comprised patients from the rural area of district Gadap and poor urban dwellers of district Lyari. The Western literature has less quantitative data on Hepatitis B and C-related health issues; however, the emerging immigrant populations from endemic zones such as Asia and Africa-owing to the insurgency, terrorism, and asylum to the west-have contributed to the increased prevalence of affected males in Canada, from 0.8% to 1%, as suggested by Sonal *et al*. [15].

In our study, the frequency for Hepatitis C and Hepatitis B was found to be 21.3% and 3.8%, indicating a fourfold increase for Hepatitis C. This could be probably due to the Expanded Program on Immunisation (EPI), administrating Hepatitis B vaccine in newborns providing some protection against virus transmission and lifelong immunity. However, unhealthy practice and lack of awareness are contributing significantly to the increase in Hepatitis C, as the virus has a long silent carrier state detected incidentally on preoperative screening by Sonal A *et al*. [13,15]. In the present study, the age group of 21-30 years is more commonly affected comparable with a study by Kausar *et al*. in pregnant females although males are more affected in our study [10]. However, Mahesh *et al*. found HBsAg and anti-HCV in the male population of age group 40-50 years predominantly, in contrast to our study where 20-30 years age group has the highest infection rate [12]. This variation could be in qualitative sample selection as cataract patients were included in their study, whereas all cases of age range from 5 to 65 years in our study. The incidence of hepatitis B can be prevented using vaccine, and hence an awareness should be raised in society for those who come for surgery and test negative so that they can be prevented and those who have encountered it should be offered treatment.

Our study population simulates a study by Ayesha *et al*. that found a 20.8% prevalence of HCV virus in young, healthy male blood donors [3]. This preoperative screening with comparable results by Paryal *et al*. is alarming, and cheap, rapid screening in preoperative time for all surgical procedures at private and public sector institutes should be recommended for enhanced safety precautions and directional treatment for carriers [11,13]. The commonest risk factor in our study was found to be parenteral injection (40.2%) followed by past surgical procedures, comparable to the local study by Rafique *et al*. as well as the study by Achakzai *et al*. involving healthy and susceptible individuals [2,16]. Also, Khatoon *et al*. study based on the rural population identified a similar frequency of risk factors at a rural hospital in India [14].

Our suggestion for this major health issue is in agreement with a study from Eastern Europe by Ganczak *et al*. for making screening policy in preoperative time for blood-borne viral transmission of the disease by rapid kit test or ELISA, to curtail risk factors in the community and treat the silent carriers [17]. Due to the lack of resources, preoperative screening was done by rapid kit test (ICT); seropositive patients were directed for ELISA and further treatment, although Ijaz *et al*. found no statistical difference in different ICT kit results with the gold standard ELISA test conducted for HBsAg and anti-HCV [18]. Therefore, this study might not be immune to practice bias.

## Conclusions

HCV was found to be the most common virus in preoperatively screened surgery patients in our study. However, parenteral abuse was found to be the most frequent risk factor in these surgical patients. The age group that tested mostly positive for HCV was young patients (21-30 years). Perhaps, for the prevention of these silent cases in the community that are detected while undergoing workup for surgery, screening is an important investigative tool that can help implement the treatment of these carrier cases.
